# Taxonomic and Biogeographic Misconceptions in Qinling Lenok (*Brachymystax tsinlingensis*): Reassessing Zhou et al. (2025) Species Distribution Models

**DOI:** 10.1002/ece3.72386

**Published:** 2025-10-23

**Authors:** Jigang Xia, Linghui Su, Yue Li, Ping Li, Youjin Hao, Shuangxi Li, Manli Zheng, Yahui Zhao

**Affiliations:** ^1^ Fish Ecology and Conservation Research Center Chongqing Normal University Chongqing China; ^2^ Laboratory of Evolutionary Physiology and Behavior, Chongqing Key Laboratory of Conservation and Utilization of Freshwater Fishes, College of Life Sciences Chongqing Normal University Chongqing China; ^3^ Power China Northwest Engineering Corporation Limited Xi'an China; ^4^ Shaanxi Taibaishan National Nature Reserve Administration Yangling China; ^5^ State Key Laboratory of Animal Biodiversity Conservation and Integrated Pest Management, Institute of Zoology Chinese Academy of Sciences Beijing China

**Keywords:** climate change, endangered salmonid, Qinling lenok *Brachymystax tsinlingensis*, species distribution models

## Abstract

The Qinling Lenok (*Brachymystax tsinlingensis*), an endangered teleost fish species endemic to China's Qinling Mountains, faces critical conservation challenges under climate change. Zhou et al. applied Maximum Entropy (MaxEnt) model to predict its potential suitable habitats under global warming and anthropogenic pressures, rightly emphasizing the urgency of protective measures (*Ecology and Evolution*, 2025, 15:e71427, https://doi.org/10.1002/ece3.71427). However, their study contains two critical flaws: taxonomic misidentification (erroneously conflating this Chinese endemic with Korean *Brachymystax* congeners) and spatial extrapolation errors (unjustified expansion of the research area beyond the species' native range), thereby compromising biogeographic accuracy and risking distributional misinterpretations. In this study, we rectifies taxonomic ambiguities by reaffirming the valid Latin binomial and strict endemicity of *Brachymystax tsinlingensis,* exposing methodological limitations in the existing distribution modeling framework. Therefore, we propose targeted enhancements—including refined species‐specific parameterization and spatially constrained climate scenarios—to improve predictive reliability for this endangered species under anthropogenic climate forcing.

## Graphical abstract

Brachymystax tsinlingensis is an endangered teleost fish species endemic to China's Qinling Mountains. Zhou et al.'s (2025) study contains two critical flaws: taxonomic misidentification and spatial extrapolation errors, thereby compromising biogeographic accuracy and risking distributional misinterpretations. We propose targeted enhancements—including refined species‐specific parameterization and spatially constrained climate scenarios—to improve predictive reliability for this endangered species under anthropogenic climate forcing.

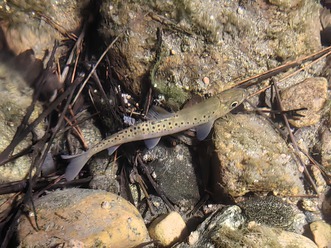

## 1 Brief Introduction to Qinling Lenok

**FIGURE 1:**
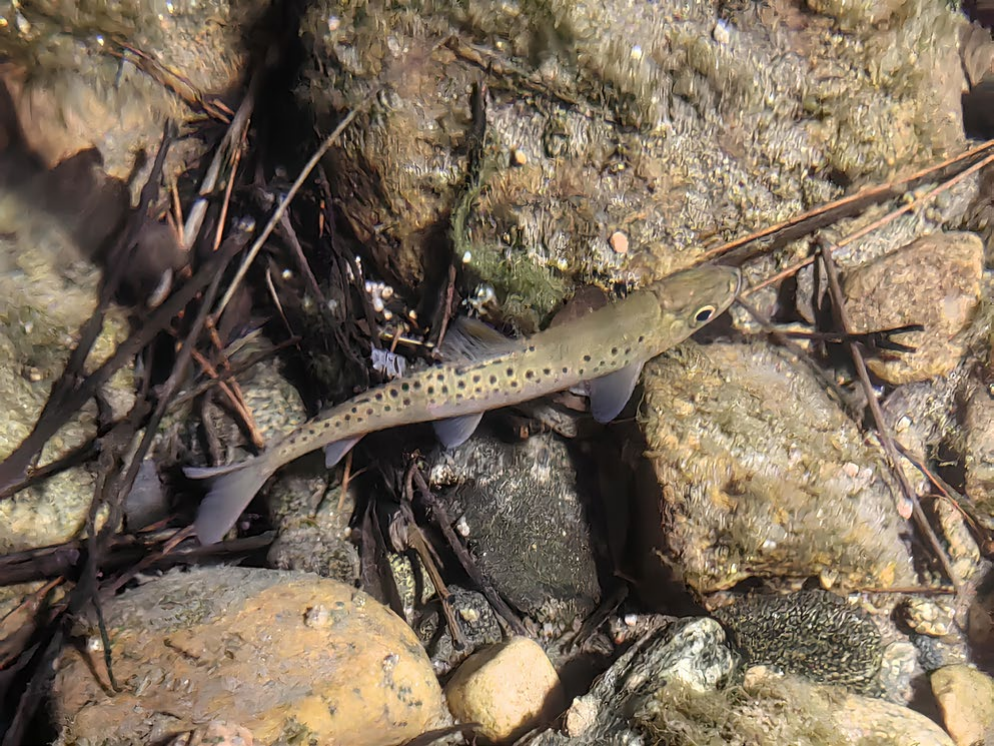
Qinling lenok *Brachymystax tsinlingensis* in its native habitats (Photographed by J. Xia).

The Qinling lenok (*Brachymystax tsinlingensis*), an endangered salmonid endemic to the Qinling Mountain region (China), has been classified as a nationally protected species since 1988 (Yue and Chen 1998; Zhao and Zhang 2009). This landlocked cold‐water fish exhibits a strong ecological preference for habitats characterized by rapid currents, clear water, and extensive gravel substrates (Figure 1). Its migratory behavior is closely tied to seasonal temperature fluctuations, with documented movements triggered by climatic shifts (Zhao and Zhang 2009; Xia et al. 2017; Tao et al. 2024). As a Quaternary glacial relict, the Qinling lenok currently faces a severe population decline. Its narrow thermal tolerance and upward shift in minimum viable elevation (from 900 to 1200 m over recent decades) position it as a critical bio‐indicator for assessing climate change impacts on freshwater ecosystems (Ren and Liang 2004; Zhao and Zhang 2009; Xia et al. 2017, 2021; Li et al. 2021; Peng et al. 2021).

Ecologically, this apex aquatic predator regulates energy flow and nutrient cycling, functioning as a flagship species for salmonid conservation in Qinling forest‐stream ecosystems (Xia et al. 2021; Deng et al. 2024; Wang et al. 2024; Wu et al. 2025). Conservation efforts include the establishment of the Qinling lenok National Nature Reserve in Longxian County, Shaanxi Province (2009) and are followed by additional habitat protection zones. These measures aim to mitigate threats from habitat fragmentation, water pollution, and climate‐driven range shifts while preserving this unique glacial relict.

## 2 Taxonomy of Qinling Lenok

The Qinling lenok (*Brachymystax tsinlingensis*) was initially described by Li in 1966 as a subspecies of *Brachymystax lenok* . However, its taxonomic validity has been the subject of ongoing debate among ichthyologists (Gao 1980; Song and Fang 1984; Song 1987; Wang 1988; Qin and Wang 1989; Ma et al. 2009; Xia et al. 2023; Li et al. 2024; Osinov 2024). Multiple taxonomic interpretations have emerged: some authorities have synonymized it with *B. lenok lenok* (Song 1987; Qin and Wang 1989), while others have aligned it with *B. tumensis* (Shedko 2001). A pivotal taxonomic reclassification occurred when Xing et al. (2015) conducted a comprehensive description of the Qinling lenok as a valid species, using type specimens and specimens collected from the type locality. Here, we propose recognizing *Brachymystax tsinlingensis* as the definitive Latin name for the Qinling lenok.

**FIGURE 2:**
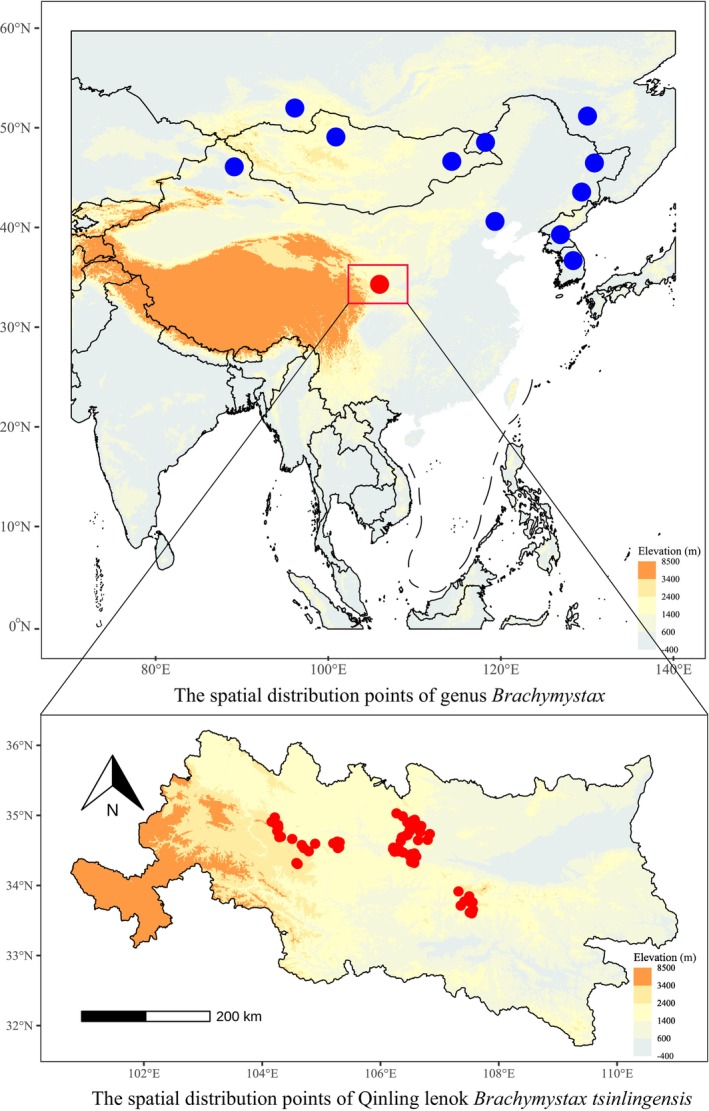
Spatial distribution of *Brachymystax* genus and *Brachymystax tsinlingensis*.

The South Korean population presents a unique paradox. While genetic analyses infer close phylogenetic affinities with *B. tsinlingensis* (Yu and Kwak 2015; Jang et al. 2017), opercular spot patterns (a key diagnostic character for species within the genus *Brachymystax*) and body elliptic markings in the Korean specimens show striking morphological congruence with *B. lenok* rather than *B*. *tsinlingensis* (Xing et al. 2015; Xia et al. 2023). This phenotypic‐genotypic discordance necessitates further investigation into the Korean population's systematic position. Furthermore, the geographic isolation of the Qinling Mountains and Korean habitats creates a significant biogeographic barrier (Figure 2), rendering conspecific status between these populations biologically implausible based on current evidence.

## 3 Spatial Distribution of Qinling Leonk

The Qinling lenok is a rare and endemic species in China, characterized by a narrow geographic range confined exclusively to cold‐water stream segments within the Qinling Mountain region (Figure 2; Zhao and Zhang 2009; Liu et al. 2013; Xing et al. 2015; Xia et al. 2017; Zhao et al. 2025). Its distribution primarily encompasses two major river basins: Weihe tributaries of the Yellow River Basin (e.g., Shitou River, Qianhe River, and Heihe River) and Hanjiang tributaries of the Yangtze River Basin (e.g., Xushui River, Youshui River, and Ziwu River). These areas represent the southernmost global distribution range of an endemic salmonid species (Zhao and Zhang 2009; Xia et al. 2021).

Zhou et al. (2025) recently modeled the distribution change of the Qinling lenok under climate scenarios using a Maximum Entropy (MaxEnt) model. However, their analysis contains significant methodological flaws: (1) Taxonomic misassignment: they erroneously incorporated South Korea specimens (likely sharp‐snouted lenok *B. lenok* ) into *B. tsinlingensis* occurrence data. This inclusion of non‐conspecific populations violates fundamental niche modeling principles. (2) Morphogenetic distinctiveness: the Qinling lenok is morphologically and genetically unique among *Brachymystax* species, with exclusive endemicity to the Qinling ecoregion (Figure 2; Zhao and Zhang 2009; Liu et al. 2013; Xing et al. 2015; Li et al. 2021; Xia et al. 2021, 2023; Xiong et al. 2023). (3) Methodological limitations: invalid research scale (i.e., overly large study region) and irrational dataset of species occurrence points preclude biologically meaningful predictions (Soley‐Guardia et al. 2024). Consequently, Zhou et al.'s (2025) distribution predictions are fundamentally compromised by taxonomic and methodological inaccuracies.

## 4 Model Suitability for Qinling Lenok Distribution Prediction

Species distribution models (SDMs) establish a quantitative relationship between environmental variables and species occurrence data. As primary tools for modeling range dynamics, SDMs provide critical baselines for conservation planning (Elith and Graham 2009; Guo et al. 2020; Zurell et al. 2020; Kong et al. 2021; Lawlor et al. 2024). Despite their utility in habitat prediction, SDMs' reliability remains contingent on appropriate algorithm selection—an ongoing methodological debate (Lee‐Yaw et al. 2022; Rios et al. 2024; Velazco et al. 2024).

Zhou et al. (2025) applied the MaxEnt model to predict the potential distribution of the Qinling lenok, but their results lack credibility due to two fundamental flaws: (1) Data integrity issues: occurrence records erroneously incorporated non‐target species (South Korean *Brachymystax* populations), which artificially expanded the assumed distribution range and compromised prediction reliability (Soley‐Guardia et al. 2024). (2) Suboptimal model implementation: they neglected essential hyperparameter tuning for optimization. Substantial evidence confirms that default MaxEnt settings frequently yield poor performance (Muscarella et al. 2014; Moreno‐Amat et al. 2015; Lissovsky and Dudov 2021; Valavi et al. 2022), necessitating parameter calibration to identify optimal configurations that reduce overfitting (Radosavljevic and Anderson 2014; Morales et al. 2017; Zhao et al. 2022). Extensive studies confirm that optimized MaxEnt implementation achieves significantly enhanced predictive accuracy and generates ecologically realistic species distribution maps (Bowen and Stevens 2020; Holder et al. 2020; Zhao et al. 2022). Therefore, Zhou et al.'s (2025) untuned model cannot support robust conservation inferences for this endangered endemic species.

## 5 Concluding Remarks and Strategic Recommendations

The study by Zhou et al. (2025) merits commendation for its timely emphasis on the urgent conservation status of *B. tsinlingensis*—an Endangered salmonid species endemic to the Qinling Mountain range. However, the article exhibits critical taxonomic misidentification by conflating this unique Chinese endemic with the Korean *Brachymystax* congeners. This fundamental error propagates through the study's methodology, resulting in three key methodological flaws: (1) Misapplied species distribution models (SDMs) based on extralimital occurrence data. (2) Geographically inconsistent research boundaries extending beyond the species' native range, and (3) Ecologically invalid climate variable correlations.

To advance future conservation research on this critically endangered species, we propose the following strategic interventions: (1) Prioritize consistent application of the approved binomial nomenclature *Brachymystax tsinlingensis* for Qinling lenok across all scientific literature, (2) Establish standardized protocols for model selection optimization and regional‐scale parameter calibration in SDMs applications, (3) Prioritize integration of empirical field population data with laboratory‐based physiological tolerance thresholds of Qinling lenok to enhance model fitting robustness, and (4) Implement a multi‐dimensional reassessment framework to quantify climate change impacts on extant and future habitat suitability of Qinling lenok.

## Author Contributions

**Jigang Xia:** conceptualization (lead), funding acquisition (lead), project administration (lead), supervision (lead), writing – original draft (lead), writing – review and editing (lead). **Linghui Su:** writing – original draft (supporting). **Yue Li:** writing – original draft (supporting). **Ping Li:** conceptualization (supporting), writing – review and editing (supporting). **Youjin Hao:** writing – review and editing (equal). **Shuangxi Li:** conceptualization (supporting), writing – review and editing (supporting). **Manli Zheng:** conceptualization (supporting), writing – review and editing (supporting). **Yahui Zhao:** conceptualization (equal), writing – review and editing (lead).

## Conflicts of Interest

The authors declare no conflicts of interest.

## Data Availability

No data were directly generated in the production of this letter.
